# Functional and Expression Analyses of the *Pneumocystis MAT* Genes Suggest Obligate Sexuality through Primary Homothallism within Host Lungs

**DOI:** 10.1128/mBio.02201-17

**Published:** 2018-02-20

**Authors:** S. Richard, J. M. G. C. F. Almeida, O. H. Cissé, A. Luraschi, O. Nielsen, M. Pagni, P. M. Hauser

**Affiliations:** a Institute of Microbiology, Lausanne University Hospital, Lausanne, Switzerland; b UCIBIO-REQUIMTE, Faculdade de Ciências e Tecnologia, Universidade Nova de Lisboa, Caparica, Portugal; c Department of Biology, University of Copenhagen, Copenhagen, Denmark; d Vital-IT Group, SIB Swiss Institute of Bioinformatics, Lausanne, Switzerland; Albert Einstein College of Medicine

**Keywords:** complementation, fission yeast, RT-PCR, gene expression, heterologous gene expression

## Abstract

Fungi of the genus *Pneumocystis* are obligate parasites that colonize mammals’ lungs and are host species specific. *Pneumocystis jirovecii* and *Pneumocystis carinii* infect, respectively, humans and rats. They can turn into opportunistic pathogens in immunosuppressed hosts, causing severe pneumonia. Their cell cycle is poorly known, mainly because of the absence of an established method of culture *in vitro*. It is thought to include both asexual and sexual phases. Comparative genomic analysis suggested that their mode of sexual reproduction is primary homothallism involving a single mating type (*MAT*) locus encompassing plus and minus genes (*matMc*, *matMi*, and *matPi*; Almeida et al., mBio 6:e02250-14, 2015). Thus, each strain would be capable of sexual reproduction alone (self-fertility). However, this is a working hypothesis derived from computational analyses that is, in addition, based on the genome sequences of single isolates. Here, we tested this hypothesis in the wet laboratory. The function of the *P. jirovecii* and *P. carinii matMc* genes was ascertained by restoration of sporulation in the corresponding mutant of fission yeast. Using PCR, we found the same single *MAT* locus in all *P. jirovecii* isolates and showed that all three *MAT* genes are often concomitantly expressed during pneumonia. Extensive homology searches did not identify other types of *MAT* transcription factors in the genomes or *cis*-acting motifs flanking the *MAT* locus that could have been involved in *MAT* switching or silencing. Our observations suggest that *Pneumocystis* sexuality through primary homothallism is obligate within host lungs to complete the cell cycle, i.e., produce asci necessary for airborne transmission to new hosts.

## IMPORTANCE

Fungi of the genus *Pneumocystis* colonize the lungs of mammals. In immunosuppressed human hosts, *Pneumocystis jirovecii* may cause severe pneumonia that can be fatal. This disease is one of the most frequent life-threatening invasive fungal infections in humans. The analysis of the genome sequences of these uncultivable pathogens suggested that their sexual reproduction involves a single partner (self-fertilization). Here, we report laboratory experiments that support this hypothesis. The function of the three genes responsible for sexual differentiation was ascertained by the restoration of sexual reproduction in the corresponding mutant of another fungus. As predicted by self-fertilization, all *P. jirovecii* isolates harbored the same three genes that were often concomitantly expressed within human lungs during infection. Our observations suggest that the sexuality of these pathogens relies on the self-fertility of each isolate and is obligate within host lungs to complete the cell cycle and allow dissemination of the fungus to new hosts.

## INTRODUCTION

The genus *Pneumocystis* includes fungal species that colonize the lungs of mammals. Each of these species is specific for a single mammalian species. *Pneumocystis jirovecii* infects humans, whereas *Pneumocystis carinii* infects rats. In immunosuppressed individuals, *P. jirovecii* can turn into an opportunistic pathogen and cause severe pneumonia (*Pneumocystis* pneumonia [PCP]) that can be fatal if not treated. This disease is one of the most frequent life-threatening invasive fungal infections in humans (1). Despite numerous endeavors, culture of *Pneumocystis* species *in vitro* has remained elusive. A method of coculturing *P. jirovecii* with lung epithelial cells at the liquid-air interface was recently described (2), but it has not been established in other laboratories yet. Analysis of the *Pneumocystis* genome sequences revealed the loss of several synthesis and assimilation pathways, showing that these fungi are obligate parasites (3–6). Their requirement of thiamine and their lack of inorganic nitrogen and sulfur assimilation are hallmarks of obligate biotrophs (7), i.e., parasites that retrieve energy and compounds from host cells without killing them. Other hallmarks of obligate biotrophs present in *Pneumocystis* species include (i) the absence of massive destruction of host cells, (ii) a lack of virulence factors, (iii) having a sex life cycle within the host, and (iv) being difficult to culture *in vitro* (5, 8–10). *Pneumocystis* species were the first described obligate animal biotrophs (10).

Mainly because of the absence of an *in vitro* culture method, the cell cycle of *Pneumocystis* species is still poorly known. Since they are obligate parasites, their cell cycle takes place entirely within the host’s lungs. It would include asexual multiplication of haploid trophic cells by binary fission, as well as a sexual phase initiated by the fusion of two trophic cells of compatible mating types (11). The sexual cycle would culminate by the production of an ascus containing eight haploid daughter cells. However, quantitative experiments suggested that meiotic division might account for all of the cell multiplication that occurs during infection (12–14). These observations suggested that an asexual cycle may not occur at all or be facultative and, consequently, that sexuality is obligatory in *Pneumocystis* species. Obligate sexuality would also be consistent with the facts that (i) asci are present in the vast majority of, if not all, human infections and are used for diagnosis by staining of their wall and (ii) asci are believed to be the aerially transported particles that ensure transmission of the fungus between hosts (15, 16).

Recently, we used comparative genomics to investigate the mode of sexual reproduction of *Pneumocystis* species (17). We used sex-related genes of the Taphrinomycotina relative *Schizosaccharomyces pombe* as sequence queries to identify homologues in the *P. jirovecii* and *P. carinii* genomes. Approximately 60 of the 103 genes investigated were identified in each species, further suggesting that sexuality is part of the *Pneumocystis* cell cycle. Importantly, in each species, we identified only three candidate homologues (*matMc*, *matPi*, *matMi*) of the four *MAT* genes present in *S. pombe*. The latter genes encode transcription factors and cofactors and are responsible for sexual differentiation into plus (P) and minus (M) mating types, as well as for induction of meiosis (18). The organization of these genes in each *Pneumocystis* genome as a fusion of the *MAT* M and P loci suggested the working hypothesis that *Pneumocystis* species are primary homothallic organisms, i.e., each strain would be self-fertile and could produce asci on its own. This contrasts sharply with the closely related yeast *S. pombe*, which uses switching of mating types involving one expressed *MAT* locus and two silenced loci (secondary homothallism) (1,9).

The present work was aimed at testing the hypothesis of obligate sexuality through primary homothallism in *Pneumocystis* species. Consistent with this hypothesis, we found the same *MAT* locus in all of the *P. jirovecii* isolates investigated and frequent concomitant expression of the three *MAT* genes within human lungs during PCP. Furthermore, the function of one putative *P. jirovecii* and *P. carinii MAT* gene was ascertained by complementation in fission yeast and new *in silico* analyses of the genomes further supported primary homothallism.

## RESULTS

### The *P. jirovecii* and *P. carinii matMc* genes functionally complement an *S. pombe matMc* null strain.

**FIG 1:**
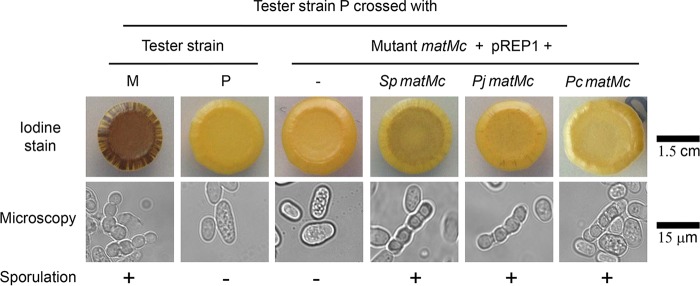
Complementation of the *S. pombe matMc* mutant by expression of the *P. jirovecii* (*Pj*), *P. carinii* (*Pc*), or control *S. pombe* (*Sp*) *matMc* gene on a plasmid. Tester strain P was crossed with various strains by using a plasmid expressing or not expressing a heterologous gene by mixing them in liquid, spotting an aliquot on the mating plate, and incubating it to allow sporulation. Complementation was assessed after 6 days of incubation at 30°C by iodine staining of spore wall starch, as well as by the presence of zygotes containing four spores (asci) upon microscopic observation. Triplicate recombinant strain isolates gave similar results.

The function of the *Pneumocystis MAT* genes remained putative, and in the absence of an *in vitro* culture method, the issue was investigated by functional complementation of *S. pombe* because it is closely related to *Pneumocystis* species and offers numerous genetic tools. The *P. jirovecii* and *P. carinii matMc*, *matMi*, and *matPi* genes were expressed separately on plasmids in the corresponding *S. pombe* mutants. The *S. pombe matMc* mutant is null because of an opal codon at the beginning of the open reading frame (ORF), whereas the *matMi* and *matPc* mutants have nonfunctional alleles because of mutations (see Materials and Methods). The empty plasmid and a recombinant plasmid expressing the corresponding wild-type *S. pombe MAT* gene were used as controls. A tester strain of the opposite mating type was crossed with each recombinant strain. Consistently in all experiments, the negative-control crosses with the tester strain of the same mating type or with the strain harboring the empty plasmid did not complement the mutant, as revealed by iodine staining and microscopic observation (e.g., *matMc* in Fig. 1). On the other hand, the positive-control crosses with the tester strain or the strain expressing the wild-type *S. pombe MAT* gene on a plasmid did complement. These experiments revealed that the *P. jirovecii* and *P. carinii matMc* genes complemented their corresponding *S. pombe* mutants. On the other hand, the *P. jirovecii* and *P. carinii matMi* and *matPi* genes did not, possibly because of lower identity with *S. pombe* proteins at the amino acid sequence level (*matMc*, 27%; *matMi*, 19%; *matPi*, 13%). The most efficient sporulation, as revealed by the intensity of the iodine staining of the colony, occurred in the cross between the tester strains, followed by the cross involving the wild-type *S. pombe matMc* gene expressed on a plasmid (Fig. 1). The low efficiency of sporulation in the latter cross might have resulted from an inadequate level of expression because of the use of the *nmt1* mutant promoter instead of the wild-type promoter. The colony from the cross involving the *P. jirovecii matMc* gene was slightly stained by iodine, whereas that from the cross involving the *P. carinii* gene remained visually negative or weakly positive (light staining might be present at the top right border of the colony). The microscopic observations correlated with the iodine staining and the *P. carinii* gene were clearly positive by this means (Fig. 1). The proportion of zygotes containing four spores in the cell population was determined for each cross by using a counting chamber under the microscope (Table 1). The values obtained correlated roughly with the intensity of iodine staining, with that of *P. carinii matMc* being the lowest. These results demonstrated that expression of the *P. jirovecii* or *P. carinii matMc* gene rescued the function of the *S. pombe* transcription factor *matMc* null mutant, ascertaining the function of these genes. This conclusion strongly suggested that the *Pneumocystis matPi* and *matMi* genes are also *MAT* genes because of their significant similarity and synteny to the *S. pombe MAT* genes (17).

**TABLE 1:** Proportions of zygotes with four spores in *matMc* complementation assay crosses

Tester strain P crossed with^a^	No. of:		% of zygotes with 4 spores	% SD between crosses
	Expt	Cells		
Tester strain M	3	1,088	3.7	0.9
*matMc* mutant + pREP1	3	1,592	0	
*matMc* mutant + pREP1 + *S. pombe matMc*	3	971	2.8	0.2
*matMc* mutant + pREP1 + *P. jirovecii matMc*	3	921	1.3	0.4
*matMc* mutant + pREP1 + *P. carinii matMc*	3	1,847	0.9	0.2

### The same single *MAT* locus is present in all *P. jirovecii* isolates.

**FIG 2:**
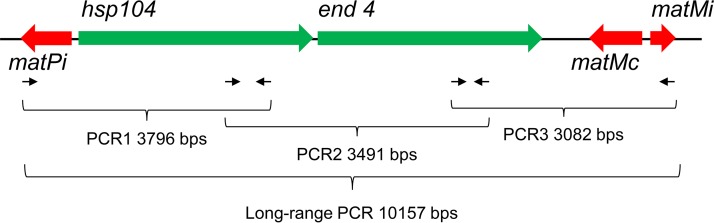
Structure of the single *MAT* locus of *P. jirovecii* corresponding to a fusion of loci P and M. The approximate locations of the primers used to confirm the presence of the locus in several isolates are shown. As in *S. pombe*, the gene encoding the huntingtin-interacting protein (*end4*) located between the *MAT* genes might be essential under most conditions, whereas the gene encoding a heat shock protein (*hsp104*) is probably not. The synteny of these genes, as well those flanking the *MAT* locus, is fully conserved in *P. carinii* and *P. murina* (17).

**FIG 3:**
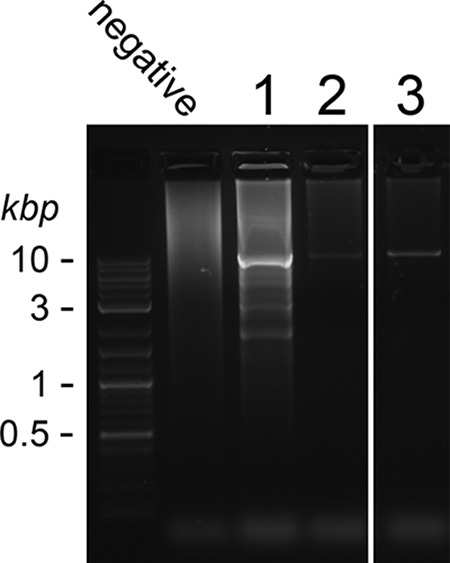
Amplification of the single *MAT* locus from three *P. jirovecii* isolates by long-range PCR. PCR analysis was performed with DNA extracted from BAL fluid samples from 11 patients with PCP; 3 are shown here as examples. The identity of the relatively pure PCR product (10,157 bp) from patient 3 was confirmed by sequencing its ends.

The three *P. jirovecii MAT* genes were clustered in a region spanning about 10 kb on a single contig of the genome sequence. Primary homothallism implies that all isolates harbor the same *MAT* locus. To investigate the issue, we used long-range PCR with primers located at the ends of the *MAT* locus, as well as PCRs with internal primers generating three overlapping products covering the whole locus (Fig. 2). Six of the 11 PCP patients analyzed were positive by long-range PCR, and 3 were positive by the three overlapping PCRs (Fig. 3; Table 2). The five patients negative by long-range PCR presented a lighter fungal load than the other patients, as revealed by real-time PCR targeting the mitochondrial 26S rDNA (*mt26S*). Long-range PCR is notoriously demanding, so that it was not surprising that it required a substantial fungal load to be positive. These observations demonstrated that the same fused *MAT* locus is present in all *P. jirovecii* isolates, which is consistent with primary homothallism as a mode of sexual reproduction.

**TABLE 2:** Amplification of the *P. jirovecii MAT* locus and *MAT* transcripts from BAL fluid samples from 11 patients with pneumonia^a^

Patient	PCR		RT-PCR			
	*mt26S* (no. of copies/ml [10^6^])	*MAT* locus	*matMc*	*matMi*	*matPi*	*β-tub*
1	+ (1,111)	+	+	+	+	+
2	+ (233)	+	−	−	−	−
3	+ (89)	+	−	+	−	+
4	+ (23)	+	+	+	+	+
5	+ (18)	+	+	+	+	+
6	+ (3)	+	−	−	+	+
7	+ (0.7)	−	+	+	+	+
8	+ (0.25)	+^b^	−	−	+	+
9	+ (0.15)	+^b^	−	−	−	−
10	+ (0.03)	+^b^	−	−	−	−
11	+ (0.01)	−	+	+	+	+

a +, positive PCR result; −, negative PCR result. The RT-PCR used to amplify the three *MAT* genes included a seminested procedure. The PCR used to amplify the *mt26S* gene included a real-time procedure (see Materials and Methods).

b Three overlapping PCRs were used (see text).

### The *P. jirovecii MAT* genes are often expressed concomitantly during infection within human lungs.

**FIG 4:**
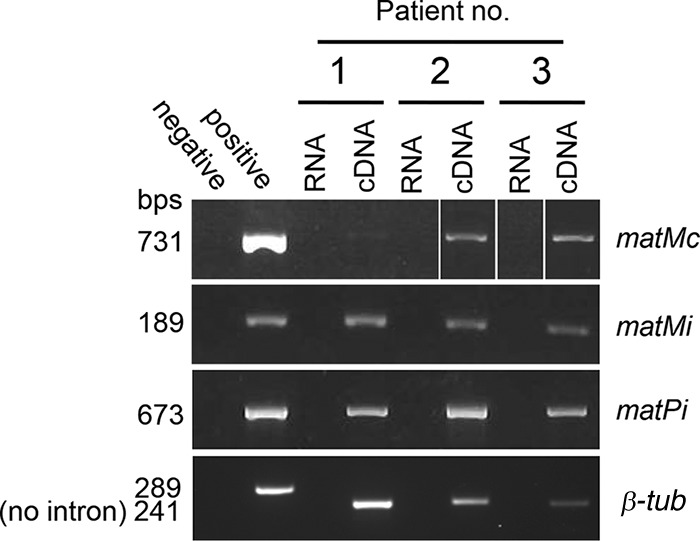
Amplification of the *MAT* and *β-tub* mRNAs of three *P. jirovecii* isolates by RT-PCR. RT-PCR analysis was performed with cDNAs obtained from BAL fluid samples from 11 patients with PCP; 3 are shown here as examples. Random amplification of the cDNA proved to be necessary to obtain PCR products. The PCR products were of the expected sizes shown next to the bands. The positive control was genomic DNA extracted from another BAL fluid sample. The *matMc* PCR products of patients 2 and 3 were obtained during an experiment other than that during which the *matMc* PCR product of patient 1 was obtained. The identities of the PCR products were confirmed by sequencing.

The *Pneumocystis MAT* loci do not present sequence motifs involved in silencing, such as the repeats resembling those of centromeres observed in *S. pombe* (*cenH*) or the actual telomere in close proximity as in *Saccharomyces cerevisiae* (17, 20). Consequently, expression of all three *P. jirovecii MAT* genes was expected to occur during PCP to ensure mating and maturation of the asci because the latter are most often, if not always, present. To investigate this issue, we used reverse transcriptase PCR (RT-PCR) analysis of total RNAs extracted from bronchoalveolar lavage (BAL) fluid samples from the 11 patients with PCP that we also investigated for the presence of the *MAT* locus (as described in the previous section). Proper RT-PCR from the BAL fluid specimens was assessed by (i) a negative PCR result with RNAs not reverse transcribed and (ii), given that the *Pneumocystis MAT* genes do not include introns, absence of the intron from the PCR product of the unrelated gene for β-tubulin (*β-tub*; Fig. 4). Of 11 patients, 5 were positive for all three *MAT* genes, 3 were positive only for one *MAT* gene, and 3 were negative for all *MAT* genes (Table 2). The latter patients were also negative for *β-tub*, suggesting that RNA degradation could have occurred during the uncontrolled period between the collection of a sample and its arrival at our laboratory. The three patients positive for only one *MAT* gene may reflect low expression of *MAT* genes. This might be due to the collection of BAL fluid at a late stage of infection, i.e., after the peak of *MAT* gene expression. Consistent with their expression, two or three potential TATA boxes matching that of 7 bp described by Bucher (21) were identified between 6 and 108 bp upstream of the start codons of the ORFs of all the *P. jirovecii* and *P. carinii MAT* genes (see Fig. S1 in the supplemental material). These observations suggested that concomitant expression of the three *MAT* genes occurs in human lungs at some stage of PCP and leads to ascus production. This is consistent with primary homothallism as the mode of reproduction and suggests that sexuality is obligatory during the infection of host lungs.

10.1128/mBio.02201-17.1 FIG S1  Potential TATA boxes upstream of *P. jirovecii* and *P. carinii MAT* genes. The potential TATA boxes were identified by visual inspection by matching that described by Bucher (21). They are shown with an arrow oriented toward the ORF, and their distances from the start codon of the ORF are shown in base pairs. Download FIG S1, PDF file, 0.04 MB. Copyright © 2018 Richard et al. 2018 Richard et al. This content is distributed under the terms of the Creative Commons Attribution 4.0 International license.

### Absence of *MAT*α*1* and amphipathic alpha-helix *MAT* transcription factors in *Pneumocystis* genomes.

*S. pombe* harbors *MAT* transcription factors with homeobox or high-motility-group (HMG) DNA binding domains. Consequently, our searches using *S. pombe MAT* genes as sequence queries (17) could not identify *MAT* transcription factors with *MAT*α*1* or amphipathic alpha-helix domains that are present in ascomycetous subphyla other than Taphrinomycotina, such as Saccharomycotina and Pezizomycotina (19). Extensive homology searches using tBLASTn involving bait sequences recruited through InterPro references did not detect any such transcription factors in the *P. jirovecii* and *P. carinii* genomes or in that of *Pneumocystis murina*, the species that infects mice. This observation further suggested that, consistent with primary homothallism, the *MAT* locus we identified is the only one present in *Pneumocystis* genomes.

### Absence of *cis*-acting sequences flanking the *MAT* locus in *Pneumocystis* genomes.

*S. pombe* harbors *cis*-acting sequences of 57 to 135 bp flanking each of its three *MAT* loci that are involved in switching of the locus expressed by homology recombination, as well as in the silencing of two *MAT* loci (20). *cis*-acting sequences flanking *MAT* loci are also present in other secondary heterothallic fungi (22), as well as in the recently described secondary homothallism based on inversion of the loci (23). We extensively compared the regions flanking the *Pneumocystis MAT* genes to each other with the help of local and global alignment tools (24), as well as that of Dotlet for visual appraisal of the distribution of local similarity (25). No such *cis*-acting sequences were identified within the three *Pneumocystis* genomes. This observation further suggested that the mode of reproduction of *Pneumocystis* species is primary homothallism and not secondary homothallism.

## DISCUSSION

Sexuality is believed to play a crucial role in the life cycle of a number of human microbial pathogens, e.g., *Plasmodium*, *Cryptococcus*, and *Candida* species, for generating genetic diversity and maintaining virulence (26–29). In the present study, we studied this process in the fungal obligate parasites of the genus *Pneumocystis*. We performed functional, structural, expression, and *in silico* genomic analyses that support the hypothesis that primary homothallism is their mode of sexual reproduction. Our observations also suggest that sexuality is obligatory within host lungs during infection to complete the cell cycle, i.e., to produce asci, which are necessary to allow dissemination of the fungus to new hosts by the air route.

### Primary homothallism of *Pneumocystis* species.

Three of our observations are strongly suggestive of primary homothallism in *Pneumocystis* species, (i) the presence of the same *MAT* locus in all *P. jirovecii* isolates investigated, (ii) the lack of other *MAT* transcription factors in the genomes and of any sequences used for switching or silencing, and (iii) the frequent concomitant expression of the three *MAT* genes during PCP. Primary homothallism is thought to be advantageous for human microbial pathogens such as *Candida*, *Cryptococcus*, and *Aspergillus* species (27–29). It would alleviate the need to find a compatible partner, and although it involves a single strain, it would avoid accumulating deleterious mutations, as well as increase genetic diversity and virulence (30). However, the exact mechanisms involved in *Pneumocystis* primary homothallism remain to be understood. The absence of the *matPc* transcription factor suggests that only M cells could be present in *Pneumocystis* populations. However, the genes coding for the receptors of both M and P factors are present in *Pneumocystis* genomes (*map3*, *mam2*) (17), suggesting that cells of both mating types are produced. The two transcription factors present in the *MAT* locus (*matPi* and *matMc*) are probably sufficient to trigger mating. Thus, the *MAT* pathways may be wired differently from those of *S. pombe* since *matPi* induces meiosis in the latter fungus but not mating (18). A caveat to the hypothesis of primary homothallism is that most, if not all, *P. jirovecii* infections are polyclonal, i.e., due to several strains (31), including all of those we analyzed in the present study (not shown; determined as described in reference 3,2). Accordingly, an alternative hypothesis is that each strain produces only, or mostly, M or P cells. This could occur by the expression at a higher level or exclusively one of the two transcription factors mentioned above through transcription regulation or by an unknown mechanism. Such a strategy might further increase genetic diversity by inducing outbreeding and would constitute a previously undescribed mode of secondary homothallism. Analyses at the gene expression level are required to characterize *Pneumocystis* sexuality further.

### Obligate sexuality of *Pneumocystis* species during infection.

Obligate sexuality is strongly suggested by our findings that (i) all three *MAT* genes are often concomitantly expressed during human infections and (ii) the *MAT* locus does not include any silencing sequence motifs. Obligate sexuality would be compatible with the hypothesis that the asci are the transmission particles (15, 16). The production of asci would allow dissemination to new hosts and thus survival of the fungus, which renders it obligate. The necessity of sexuality might also ensure antigenic variation through the frequent recombination events that occur between the genes encoding surface antigens localized within the subtelomeres (32). Indeed, the bouquet of telomeres formed during meiosis is believed to favor ectopic recombinations at subtelomeres (33, 34). Interestingly, dissemination by asci may also ensure the polyclonality of the infections, i.e., their descent from several cells, which might be of importance. If sexuality is indeed obligate, then it is likely that it also occurs in colonized hosts without overt disease because colonized humans are thought to constitute a source of the infection (35, 36). Obligate sexuality does not imply that asexual reproduction of the trophic forms does not occur. Indeed, multiplication of the trophic forms could be facultative, depending on the conditions encountered. Treatment with echinocandins eliminates asci but not the trophic forms from infected rodents, suggesting that asexual multiplication of the trophic forms may persist under these conditions (15). Antipneumocystis prophylaxis has been shown to reduce the proportion of asci within the population (37). Two other cases harboring few asci were reported (38), but it remained unclear if they were due to prophylaxis or an unknown factor. It is also possible that asexual reproduction is preponderant in early stages of infection, when growth is relatively fast, until the niche is filled with *Pneumocystis* cells. When nutriments become limited, asexual reproduction might be followed by mating and sporulation.

### Conclusions.

Our observations suggest that the sexuality of *Pneumocystis* species occurs by primary homothallism (self-fertility of each strain) and that it is obligatory within the host’s lungs during infection. Obligate sexuality may allow completion of the cell cycle, i.e., production of asci that would be the airborne particles that allow transmission of the fungus to new hosts, and thus its survival. Further work is needed to understand the mechanisms of this reproduction, as well as the relative importance of the sexual and asexual phases.

## MATERIALS AND METHODS

### Strains and growth conditions.

Eg2772 is a haploid *S. pombe* strain with a null mutation due to an opal codon at the seventh position of the ORF of the gene encoding the *matMc* HMG transcription factor (*h*^−^ *mat1*-*Mc*-*H1*::*ura4*^+^ *leu1 ura4*-*D18 ade6*-*M210*). It is an unpublished derivative of strain Eg575 (20). Eg903 is a haploid *S. pombe* strain with a nonfunctional mutated allele of the *matPi* homeobox transcription factor (*h90 mat2*-*Pm*-*B102 ura4*-*D18 leu1*-*32*) (39). Eg904 is a haploid *S. pombe* strain with a nonfunctional mutated allele of the *matMi* transcription cofactor (*h90 mat3*-*Mm*-*B406 ura4*-*D18 leu1*-*32*) (39). In the present study, these strains are named the *matMc*, *matPi*, and *matMi* mutants, respectively. They have impaired sporulation upon mating and produce zygotes lacking spores inside (Fig. 1). Eg545 and Eg545 are haploid *S. pombe* tester strains, respectively, *h*^*+*^ and *h*^*−*^ (20).

*S. pombe* strains were grown for maintenance and experiments at 30°C in liquid or on solid (2% [wt/vol] agar; Difco) Edinburgh minimal medium EMM, which consists of 14.7 mM C_8_H_5_KO_4_, 15.5 mM Na_2_HPO_4_, 93.5 mM NH_4_Cl, 2% glucose, 1× salt stock (50× concentrated salt stock: MgCl_2_ ⋅ 6H_2_O at 0.26 M, CaCl_2_ ⋅ 2H_2_O at 5 mM, KCl at 0.67 M, Na_2_SO_4_ at 4.1 mM), 1× vitamin stock (1,000× vitamin stock is sodium pantothenate at 81 mM, nicotinic acid at 81 mM, inositol at 4.2 mM, and biotin at 41 μM), and 1× mineral stock (10,000× mineral stock is H_3_BO_3_ at 81 mM, MnSO_4_ at 33 mM, ZnSO_4_ ⋅ 7H_2_O at 14 mM, FeCl_3_ ⋅ 6H_2_O at 7.4 mM, molybdic acid at 0.32 mM, KI at 6 mM, CuSO_4_ ⋅ 5H_2_O at 1.6 mM, and citric acid at 48 mM [molybdic acid induces opaqueness, which is eliminated by citric acid]). The medium was complemented at 225 mg/liter with each compound necessary to supplement autotrophy.

### Source and cloning of *MAT* gene sequences.

The coordinates of the *P. jirovecii matMc*, *matMi*, and *matPi* gene sequences within the genome are, respectively, LFWA01000009.1:221076.220324 (locus T551_02162), LFWA01000009.1:221185.221394 (no locus defined), and LFWA01000009.1:211902.211243 (locus T551_02159). Those of the *P. carinii matMc*, *matMi*, and *matPi* gene sequences are, respectively, LFVZ01000013.1:80958..80260 (locus T552_02831), LFVZ01000013.1:81265..81480 (no locus defined), and LFVZ01000013.1:72050..71358 (locus T552_02829). The ORFs of all of these *MAT* genes harbor no introns and were thus directly amplified by PCR from the genomic DNA extracted from a BAL fluid specimen from an HIV-positive patient with PCP (see PCR amplification section) or from *P. carinii* genomic DNA (rat 876/4-1997, kindly provided by the late A. E. Wakefield, University of Oxford). Each PCR product was cloned into the vector pREP1 (40) downstream of a promoter, ensuring strong constitutive expression of the heterologous gene in *S. pombe*. The *S. pombe matMc*, *matMi*, and *matPi* genes cloned in pREP1 were previously described (respectively, plasmids pSK138 [41], pUS130, and pON656 [42]). Each recombinant plasmid was introduced into *Escherichia coli* DH5α competent cells obtained as described by Chung and Miller (43), and minipreparations of plasmid DNA were made as described by Birnboim and Doly (44). Each plasmid was then introduced into the corresponding *S. pombe* mutant by transformation as described previously (45).

### Complementation tests.

A small amount of cells of the tester strain grown on EMM was resuspended in 0.1 ml of EMM, which was then divided into 10 equal aliquots. A small amount of cells of the strain under test grown on EMM was added to one of the aliquots, and the mixture was well mixed and then deposited on EMM, which was incubated at 30°C. After 6 days, asci were observed under a phase-contrast microscope and counted in a Neubauer counting chamber (0.0025 mm^2^, 0.1-mm depth). To stain the starch present in the spore wall, iodine crystals (Sigma) were placed in the lid of a petri dish under a chemical hood and the plates carrying colonies issued from the crosses were exposed to the crystals for 5 to 10 min, depending on the staining intensity obtained. The crosses gave similar results to those shown in Fig. 1 by the method of crossing growth lines on plates (results not shown).

### PCR amplification.

To avoid contamination, PCRs were set up and analyzed in separate rooms and negative controls were systematically run. PCRs were performed with genomic DNA extracted from 0.2 ml of BAL fluid from PCP patients with the QIAamp DNA blood kit (Qiagen, Basel, Switzerland). Each reaction began with denaturation for 3 min at 94°C, followed by 40 cycles of 30 s at 94°C, 30 s at the annealing temperature, and 0.5 to 11 min at 72°C (68°C for Kappa polymerase; primers and parameters specific to each PCR are shown in Table S1). The reaction ended with 10 min at 72°C (4 min at 72°C for Kappa polymerase). To verify the identities of the PCR products, sequencing of both strands was performed with the two primers used for amplification, as well as the BigDye Terminator DNA sequencing kit and an ABI PRISM 3100 automated sequencer (both from PerkinElmer Biosystems).

10.1128/mBio.02201-17.2 TABLE S1  Conditions used for PCR amplification in this study. Download TABLE S1, PDF file, 0.03 MB. Copyright © 2018 Richard et al. 2018 Richard et al. This content is distributed under the terms of the Creative Commons Attribution 4.0 International license.

The real-time PCR specific for *P. jirovecii* amplifies a 77-bp fragment of the mitochondrial 26S rDNA (*mt26S*), a gene present in multiple copies per cell. Total DNA was extracted from 200 μl of thawed BAL fluid and finally eluted in 100 μl with the MagNA Pure LC robot and DNA isolation kit I (both from Roche). A Pipetting Tecan EVO 150 robot (eight channels) was used to prepare a reaction mixture containing ABI TaqMan universal PCR master mix, forward primer TGCAAAGTACTCAGAAGAATTGTGGTA, reverse primer TTCGCAGAAAACCAGCTATATCCT, and minor groove binding probe 6-carboxyfluorescein–CCGATTTGTATTTCACTAT–Black Hole Quencher 1. Each PCR included a 15-μl reaction mixture and 5 μl of extracted DNA. Real-time PCR was performed with TaqMan 7900 (Applied Biosystems). After 2 min at 50°C and 10 min at 95°C, 45 cycles of 15 s at 95°C and 1 min at 60°C were performed. The specificity of the PCR was assessed by the absence of amplification with genomic DNA of microorganisms commonly found in BAL fluids. Its sensitivity was determined by amplification of successive dilutions of a control plasmid containing the *P. jirovecii* target sequence. The limit of detection was 10 copies of the target per reaction mixture. Conversion of the *C*_*T*_ value to the target concentration was obtained by using a calibration curve obtained by amplification of successive dilutions of the control plasmid, which was dosed with a NanoDrop ND-1000 spectrophotometer. Absence of inhibition was tested for by using a reaction mixture containing both the test DNA and 1,000 copies of the control plasmid. Each sample was analyzed in duplicate.

### RT-PCR amplification.

RT-PCRs were performed with cDNAs synthesized from total RNAs that were extracted with the RiboPure yeast kit (Ambion) from 1 ml of BAL fluid from a patient with PCP. The BAL fluids were previously preserved at −80°C in RNAlater (Ambion) immediately upon receipt. cDNAs were synthesized from 8 μl of an RNA preparation with the Qiagen REPLI-g WTA Single Cell kit involving random amplification. cDNA was then purified by LiCl-ethanol precipitation (Qiagen supplementary protocol) in the presence of 5 μg of glycogen (stock 20 mg/ml; Thermo Scientific). Random amplification of cDNA included in the kit proved to be necessary for detection of the low-abundance *P. jirovecii* cDNAs among the human ones. The PCR procedure was as described above, and the primers and parameters specific to each PCR are shown in Table S1. A seminested procedure was used to amplify the *MAT* genes in a second round with 2 μl (0.5 μl for the positive control) of the first-round reaction product as the template.

### Bioinformatic analyses.

Potential genes encoding *MATα1* or amphipathic alpha-helix transcription factors were searched for in the *P. jirovecii*, *P. carinii*, and *P. murina* genomes (respective accession numbers LFWA01000000, LFVZ00000000, and AFWA00000000) by matching these genomes against large pools of representative bait sequences by using tBLASTn (NCBI BLAST suite, 64-bit version). These pools of bait sequences were recruited through the InterPro annotations IPR006856 (mating type protein MAT alpha 1, HMG-box) and IPR031472 (MAT1-1-2/MatA-2/Smr1 family). To avoid missing candidates because of the use of too stringent conditions, the tBLASTn searches were conducted with relaxed parameters (E value from 1E-4 down to the default value). Each match with a suitable E value (<1) was investigated by looking for preexisting annotations. If no coding sequence (CDS) annotations were available, the matched region was assessed for putative novel CDSs and their translated sequence were submitted to the InterProScan4 tool to detect the required reference signature matches.
