# Supplementary material for: Functional and Expression Analyses of the Pneumocystis MAT Genes Suggest Obligate Sexuality through Primary Homothallism within Host Lungs
Source: mBio. 2018 Feb 20;9(1):e02201-17. doi: 10.1128/mBio.02201-17 (PMC5821091; doi:10.1128/mBio.02201-17)
Supplement: FIG S1 [file mbo001183738sf1.pdf]

P. jirovecii

```
                                             -91 →
aactgtgcat gcattgtctt ttagacaaat atatacaatt ttgattaaat tcccaaaata
ttgacacgta cgtaacagaa aatctgttta tatatgttaa aactaattta agggttttat
                                 ← -14
<....matMc...<<
 l  q  a   h  m

                                                           -7 →
tgtttttaaa atgcttcaaa taaatcccag gatcccttgc cttgaaatcc ctttataaag
acaaaaattt tacgaagttt atttagggtc ctagggaacg gaactttagg gaaatatttc
                  ← -61                                ← -96
ataccaaatg cttccagtat gttttgttct gataattttg ctaataacgg cactccaact
tatggtttac gaaggtcata caaaacaaga ctattaaaac gattattgcc gtgaggttga
      >>......................matMi.........................>
        m   l  p  v   c  f  v   l  i  i  l   l  i  t   a  l  q
```

P. carinii

```
aatattcaag atgtgcatat ggcatgcttg ccccttcacc ctttacccat gtattcccta
ttataagttc tacacgtata ccgtacgaac ggggaagtgg gaaatgggta cataagggat
<.........matMc.........<<
l  y  e  l   h  a  y   p  m

ctgattttat gtgtattttt taagatatct ttattttgag cgccttgcat tagtcagtga
gactaaaata cacataaaaa attctataga aataaaactc gcggaacgta atcagtcact
    ← -38                         ← -63
aaaaaaatag acctttaccc ttctttcctt aagctttaat aagggtgcca cctttgttag
tttttttatc tggaaatggg aagaaaggaa ttcgaaatta ttcccacggt ggaaacaatc

ccttctctaa agtaaaataa ccaaatatct tctttatgtt tcttgatgta ggcagaaaaa
ggaagagatt tcattttatt ggtttataga agaaatacaa agaactacat ccgtcttttt

agatgcccaa agcacttagg ctttcttttt tgtacagcca ccttagattt ttttatccaa
tctacgggtt tcgtgaatcc gaaagaaaaa acatgtcggt ggaatctaaa aaaataggtt
            -20 →          -6 →
tctcaattaa ttgcattctt taaagcaata tatgaatgtt cttaaccatt tgtataagat
agagttaatt aacgtaagaa atttcgttat atacttacaa gaattggtaa acatattcta
                               >>..........matMi............>
                                 m  n  v   l  n  h   l  y  k
```

P. jirovecii

```
                                              -108 →
gaaaatttcg agtaaattga atgaattaga aggcaaaaag tatattttta tgttttttgg
cttttaaagc tcatttaact tacttaatct tccgtttttc atataaaaat acaaaaaacc
                                              -16 →
ggttttgagt gagttttttg ggtaaaaaaa agtaaaaaat atttctaaca aaaaagtaga
ccaaaactca ctcaaaaaac ccattttttt tcatttttta taaagattgt tttttcatct

aaatgttgtc tattgggtca ttagatgttt catgtatgga ttcagtgaat gaaaataggt
tttacaacag ataacccagt aatctacaaa gtacatacct aagtcactta cttttatcca
  >>........................matPi..........................>
    m  l   s  i  g  s   l  d  v   s  c  m   d  s  v  n   e  n  r
```

P. carinii

```
tcagcgccgt gttcgacata ttaatgggta taattgacgg ggaattataa ggtgttttgg
agtcgcggca caagctgtat aattacccat attaactgcc ccttaatatt ccacaaaacc
                              -52 →                    -29 →
aaaagaggaa ttatacagga atttataaag aaaaatatag agaaaatata aaggaattgc
ttttctcctt aatatgtcct taaatatttc tttttatatc tcttttatat ttccttaacg

tatagggaaa agagagtata atatgtcaga ggaggtatat gatgtttttt gtggggaaat
atatcccttt tctctcatat tatacagtct cctccatata ctacaaaaaa caccccttta
                         >>...............matPi................>
                           m  s   e  e  v  y   d  v  f   c  g  e
```

Fig. S1
