# Supplementary material for: Functional and Expression Analyses of the Pneumocystis MAT Genes Suggest Obligate Sexuality through Primary Homothallism within Host Lungs
Source: mBio. 2018 Feb 20;9(1):e02201-17. doi: 10.1128/mBio.02201-17 (PMC5821091; doi:10.1128/mBio.02201-17)
Supplement: TABLE S1 [file mbo001183738st1.pdf]

Table S1. Conditions for PCR amplifications.

Target, use [a]	Primer name	Primer 5'-3' nucleotide sequence	Annealing temperature (°C)	Extension time (min)	Mg^{2+} final concentration (mM)	PCR product size (bp)
Pj matMc ORF, cloning[b]	pjmatMcstaSAL<br>pjmatMcendBAM	CCCCCCGTCGACATGCATGCACAGTTAGAATTC<br>CGCGGGATCCTCATACCCACAGTGGGGCTG	58	1	3	777
Pj matMi ORF, cloning	pjmatMistaSAL<br>pjmatMiendBAM	CCCCCCGTCGACATGCTTCCAGTATGTTTTGTTC<br>CGCGGGATCCTCAATGTGGCTCAGTAGAATATAC	55	1	3	234
Pj matPi ORF, cloning	pjmatPistaSAL<br>pjmatPiendBAM	CCCCCCGTCGACATGTTGTCTATTGGGTCATTAG<br>CGCGGGATCCTTATTCCGAATTGGAAAACAC	55	1	3	718
Pc matMc ORF, cloning	pcmatMcstaSAL<br>pcmatMcendBAM	CCCCCGTCGACATGCCATATGCACATCTTG<br>CCCCGGGATCCTTATGCCCAAAGTCCCGTAG	56	1	3	720
Pc matMi ORF, cloning	pcmatMistaSAL<br>pcmatMiendBAM	CCCCCCGTCGACATGAATGTTCTTAACCATTTG<br>CGCGGGATCCTTAGAAGGAAAACCAATGG	56	1	3	237
Pc matPi ORF, cloning	pcmatPistaSAL<br>pcmatPiendBAM	CCCCCCGTCGACATGTCAGAGGAGGTATATG<br>CGCGGGATCCTTATTCAGAATTAGCGAATACC	58	1	3	715
Pj matMc ORF, first round	pjmatMcstart<br>pjmatMcend	ATGCATGCACAGTTAGAATTC<br>TCATACCCACAGTGGGGCTG	58	1	3	755
Pj matMi ORF, first round	pjmatMistart<br>pjmatMiend	ATGCTTCCAGTATGTTTTGTTC<br>TCAATGTGGCTCAGTAGAATATAC	58	1	3	212
Pj matPi ORF, first round	pjmatPistar<br>pjmatPiend	ATGTTGCTCATTGGGTCATTA<br>TTATTCCGAATTGGAAAACAC	54	1	3	696
Pj matMc ORF, second round	pjmatMcstartNEST<br>pjmatMcend	CTACCAAATGAAACAGAAAC<br>TCATACCCACAGTGGGGCTG	60	1	3	731
Pj matMi ORF, second round	pjmatMistartNEST<br>pjmatMiend	GATAATTTTGCTAATAACGG<br>TCAATGTGGCTCAGTAGAATATAC	54	1	3	189
Pj matPi ORF, second round	pjmatPistartNEST<br>pjmatPiend	TGTTTCATGTATGGATTCAG<br>TTATTCCGAATTGGAAAACAC	54	1	3	673
Pj MAT locus	pjmatPiend<br>pjmatMiend	TTATTCCGAATTGGAAAACAC<br>TCAATGTGGCTCAGTAGAATATAC	52	11	1.75	10157
Pj MAT locus, PCR1	pjmatPiend<br>pjMat-intrev1	TTATTCCGAATTGGAAAACAC<br>AATAACCTTCCTGTGCGG	54	4	3	3796
Pj MAT locus, PCR2	pjMat-intfor2<br>pjMat-intrev2	CACCAAATTTAGTTAGCAGAG<br>GTCACCATTTGGACCATC	54	4	3	3491
Pj MAT locus, PCR2	pjMat-intfor3<br>pjmatMiend	CATCGTGATATGGATTCTGC<br>TCAATGTGGCTCAGTAGAATATAC	54	4	3	3082
Pj β-tubulin region<br>Surrounding intron no.6	44<br>45	TCATTAGGTGGTGGAACGGG<br>ATCACCATATCCTGGATCCG	56	0.5	3	289/<br>241 no intron

[a] Pj, *P. jirovecii*. Pc, *P. carinii*. The proofreading high-fidelity expand polymerase (Roche Diagnostics) was used for all PCRs except the four used for amplification of the *MAT* locus which were carried out using the KAPA Long Range HotSart polymerase (KAPA biosystems).

[b] Restriction sites are underlined.
